# There is no transfer of mitochondria from donor hematopoietic cells to recipient mesenchymal stromal cells after allogeneic hematopoietic stem cells transplantation in humans

**DOI:** 10.1016/j.htct.2025.103859

**Published:** 2025-06-18

**Authors:** Natalya Sats, Vadim Surin, Tatiana Abramova, Aleksandra Sadovskaya, Nataliya Petinati, Nikolay Kapranov, Ksenia Nikiforova, Nina Drize, Luisa Karaseva, Olga Pokrovskaya, Larisa Kuzmina, Elena Parovichnikova

**Affiliations:** a Federal State Budgetary Institution Scientific Medical Research Center for Hematology of the Ministry of Health of the Russian Federation, 125167, Moscow, Novy Zykovsky Ave., 4, Russia; b Moscow University named after. M.V. Lomonosov, 119991, Moscow, GSP-1, Leninskie Gory, Russia

**Keywords:** Mesenchymal stromal cells, Hematopoietic stem cell transplantation, Mitochondria, Mitochondrial DNA

## Abstract

**Introduction:**

Multipotent mesenchymal stromal cells are progenitors of the bone marrow stromal microenvironment that support hematopoiesis. Mitochondria, which can be transferred between cells via nanotubes or extracellular vesicles, play a key role in the functions of mesenchymal stromal cells. In a murine model, donor hematopoietic stem and progenitor cells transfer functional mitochondria to bone marrow mesenchymal stromal cells of the recipient. The aim of this study was to find out whether such transfer occurs in humans after allogeneic hematopoietic stem cell transplantation.

**Methods:**

This study included nine patients with acute leukemia who received a reduced intensity conditioning regimen. Donor hematopoietic stem and progenitor cells mobilized into peripheral blood were the source of transplanted stem cells. Total DNA was isolated from bone marrow mesenchymal stromal cells of each patient before and after transplantation and their respective donors’ leukocytes. A fragment of mitochondrial DNA including the full-length control region was sequenced. The mitochondrial DNA sequence of each patient’s mesenchymal stromal cells was compared before and after the procedure and with the respective donor leukocytes.

**Results:**

Donor mitochondrial DNA was not detected in the mesenchymal stromal cells of any patient after transplantation even as trace amounts. Co-culturing donor leukocytes with intact and irradiated mesenchymal stromal cells *in vitro* did not lead to detection of donor mitochondrial DNA transfer.

**Conclusion:**

The data show that there is no mitochondrial transfer from donor hematopoietic stem and progenitor cells to recipient mesenchymal stromal cells after transplantation. Thus, the results indicate that one cannot count on improved mesenchymal stromal cell metabolism due to mitochondrial transfer. It is necessary to look for other ways to restore the stromal microenvironment.

## Introduction

Multipotent mesenchymal stromal cells (MSCs) support hematopoiesis and exhibit strong immunomodulatory properties due to the secretion of growth factors, cytokines, and extracellular vesicles. MSCs and their extracellular vesicles (MSC-EVs) are widely tested in clinical trials. For example, MSCs have been used to treat critically ill patients with sepsis,^1^ COVID-19 pneumonia,^2^ and for the prevention of graft-versus-host disease.^3^ MSC-EVs can exert immunomodulatory effects in multiple sclerosis, rheumatoid arthritis, and type 1 diabetes, and are also effective in cardiac, hepatic, and renal regeneration.^4^ MSC-EVs can deliver mRNA, miRNA, cytokines, and growth factors to target cells and some contain mitochondria that are transferred to recipient cells.^5^

The role of mitochondria is not limited to energy production: they are essential for redox balance maintenance, apoptosis regulation and metabolic programming and thus have a significant impact on cell fate and functionality. Mitochondria dysfunction disrupts tissue integrity causing a number of human diseases.^6^ Their activity is important for many facets of stem cell fate, including quiescence, proliferation and differentiation.^7^ In recent years, it has been found that mitochondria play a key role in regulating different functions of MSCs and are a putative target for various therapies.^8^

During cell division, mitochondria are transmitted from parent to daughter cells. Over the past two decades, an ever-growing body of evidence was accumulated that some cell types can export mitochondria via nanotubes^11^ or extracellular vesicles^12^ to unrelated cells in a process called horizontal mitochondrial transfer.^9^^,^^10^ Mitochondrial exchange is believed to be an essential form of intercellular signaling involved in homeostasis, stress response and immune reactions.^13^

MSCs and their mitochondria are currently in the spotlight due to their clinical relevance and therapeutic potential. MSCs have been found to donate mitochondria to vascular smooth muscle cells, neurons, lymphocytes, macrophages and other cell types, including a variety of malignant cells.^14^ In the bone marrow, stromal cells transfer mitochondria to hematopoietic progenitors as a response to acute infection, boosting their expansion and differentiation to leukocytes.^15^

On the other hand, MSCs-derived mitochondria support not only normal hematopoietic cells, but also leukemic cells, contributing to progression and therapy resistance.^16^^,^^17^^,^^18^ MSCs primed by reactive oxygen species-inducing agents actively transferred mitochondria and were able to mitigate oxidative stress in co-cultured acute lymphoblastic leukemia (ALL) cell lines and in a murine ALL model.^19^ The importance of mitochondria for this was highlighted by the fact that mitochondrial depletion or prevention of mitochondrial transfer abolished the rescue.^17^ The therapeutic efficacy of MSCs appears to be closely tied to their ability to donate Mitochondria as well as to mitochondrial activity.^20^

Despite the fact that most studies focus on their ability to donate mitochondria, MSCs can be on the receiving end of the mitochondrial transfer as well. In a murine model, donor hematopoietic stem and progenitor cells (HSPCs) transfer functional mitochondria to the recipient bone marrow MSCs after allogeneic hematopoietic stem cell transplantation (allo-HSCT).^21^ Notably, MSCs (but not other stromal or endothelial bone marrow cells) demonstrated a dramatic loss of mitochondrial mass after total body irradiation.^22^ After mitochondrial transfer, both host stromal microenvironment recovery and donor HSPC engraftment were improved, resulting in better hematopoiesis reconstitution. These findings demonstrate that donor HSPCs not only restore the hematopoietic system after allo-HSCT, but also induce and support the recovery of the irradiated microenvironment via mitochondrial transfer.

HSPC-to-MSC transfer of mitochondria was also observed after *in vitro* co-cultures; however, the efficiency was much lower, possibly because of the shorter time or the absence of *in vivo* signals triggering the mitochondrial exchange.^21^ *In vitro* co-culture of murine MSCs with both murine and human CD34^+^ HSPCs led to similar rates of mitochondrial transfer.

Reciprocal mitochondrial transfer between host and recipient HSPCs was observed in a murine model using mitochondrial-nuclear exchange mice (hybrid mice with nuclear DNA from C57BL and mitochondrial DNA from C3H/HeN strains).^23^

Studying horizontal mitochondrial transfer presents certain challenges. Most studies use Mitotracker Red (or similar potential-based dyes) as an mitochondrial marker. However, recently this approach was proven unreliable by Chen et al.^14^ The authors showed that tracking mitochondria with GFP-fused mitochondrial proteins results in the detection of mitochondrial transfer in significantly fewer cases than utilizing Mitotracker. Furthermore, staining of Mt-deficient cells convincingly proved that potential-based mitochondrial dyes are not specific enough and can lead to false-positive results.^14^

## Objective

The aim of this work was to find out whether mitochondria are transferred from transplanted HSPCs to the recipient MSCs after allo-HSCT in humans. In order to avoid potential-based mitochondrial dyes, mtDNA Sanger sequencing was used as a highly specific method suitable for low copy detection of donor mtDNA in the recipient MSCs.

## Method

### Patients

This study was approved by the ethics committee of the Federal State Budgetary Institution National Medical Research Center for Hematology of the Ministry of Health of the Russian Federation (protocol No 171 dated April 27, 2023) and the donors and patients provided written informed consent before inclusion. The samples were obtained in accordance with the Declaration of Helsinki.

The study analyzed the data of nine patients with acute leukemia before and one month after allo-HSCT (Supplementary Table 1). In all cases, hematopoietic stem cells mobilized into peripheral blood were used (total CD34^+^ cell dose >6 × 10^6^ cells/kg). The patients received a reduced intensity conditioning regimen (fludarabine, busulfan). The point of analysis after allo-HSCT was chosen as it matches the average time of donor hematopoiesis reconstitution (number of leukocytes in peripheral blood >1 × 10^3^ cells/µL).

### Experimental design

**Figure 1:**
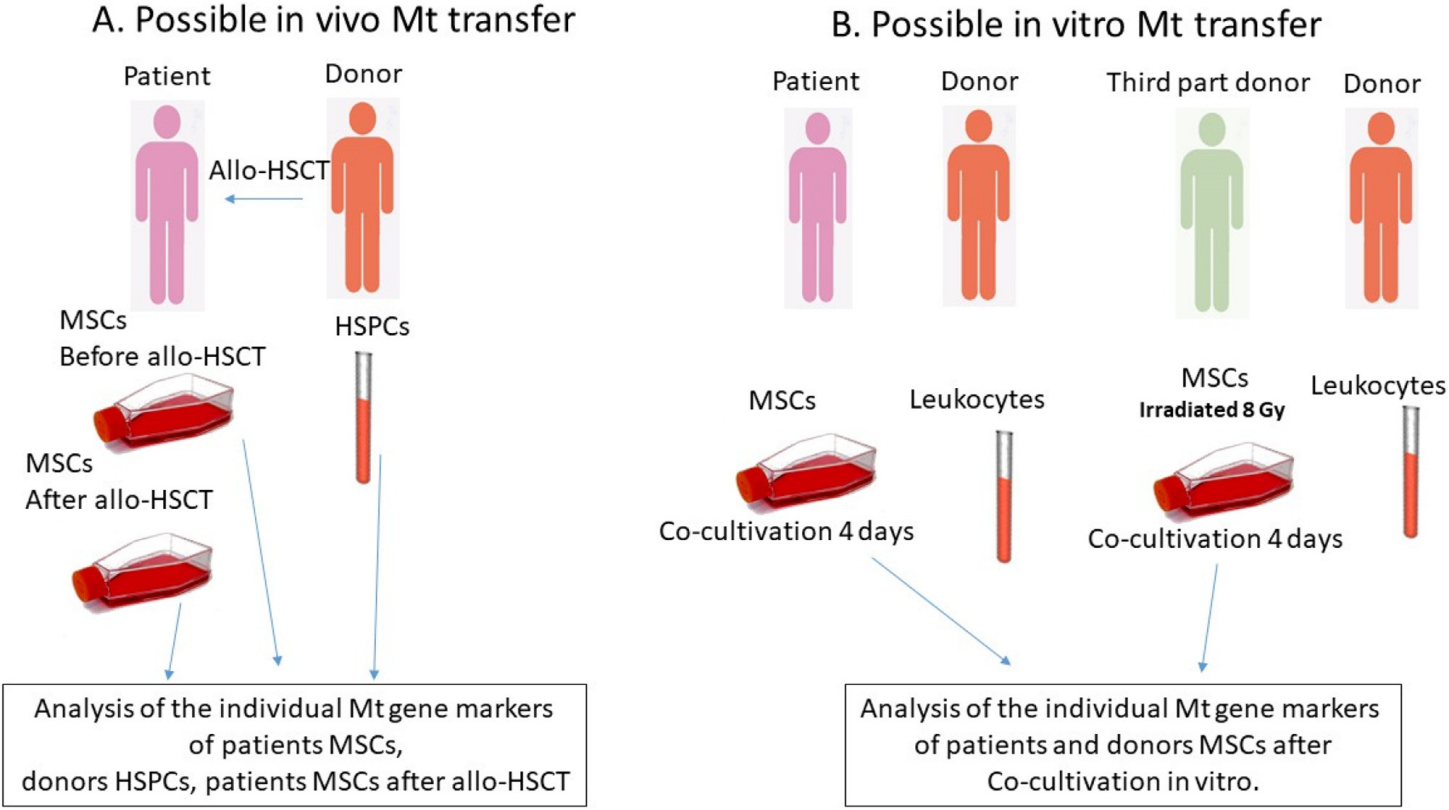
Study design outline.

To test the transfer of Mt, MSCs from patient bone marrow were studied before and one month after allo-HSCT. The patient bone marrow was obtained during a diagnostic puncture, and MSC culture was established (Figure 1A). In addition, patient or third-party donor confluent MSCs were co-cultured *in vitro* with donor CD45^+^ cells - leukocytes (10^6^- 5 × 10^6^ cells per six-well plate: Figure 1B) for four days, and then the mitochondrial composition of the MSCs was analyzed. In some experiments, third-party donor MSCs were irradiated at a dose of 8 Gy using a gamma irradiator BioBeamGM 8000 (Gamma-Service Medical, Germany) at a dose rate of 1.4 Gy/min. The irradiation regimen was chosen in order to approximate the doses used *in vivo* to the *in vitro* model.

After co-culture MSCs were washed from leukocytes and passaged two more times to avoid the mtDNA of donor leukocytes being included in the analysis.

A. Analysis for the *in vivo* mitochondrial transfer from donor hematopoietic stem and progenitor cells (HSPCs) to patient mesenchymal stromal cells (MSCs)
  The bone marrow of patients was collected prior to the allogeneic hematopoietic stem cell transplantation (allo-HSCT) procedure, and the MSCs were isolated and screened for matching MSC criteria. The individual mitochondrial gene markers were analyzed at the second passage. The donor lymphocytes were also analyzed for individual mitochondrial gene markers. The bone marrow was collected during a diagnostic procedure one month after the allo-HSCT, and the MSCs were obtained for analysis, with the mitochondrial gene markers being analyzed at the second passage.
B. Analysis for the *in vitro* mitochondrial transfer from leukocytes to MSCs
  The patient MSCs at the second passage were cultured with donor lymphocytes for a period of four days. Subsequently, the lymphocytes were removed, MSCs were washed and passaged. The mitochondrial gene markers in the MSCs were examined. In certain experiments, the MSCs from a third-party donor were exposed to a dose of 8 Gy of radiation.

### MSC isolation and culture

Bone marrow was obtained during diagnostic punctures of patients. Healthy bone marrow for MSC acquisition was obtained during exfusion for allo-HSCT. To prevent clotting, 2–7 mL of bone marrow were placed in sterile tubes with 1 mL of heparin (50 U/mL). The bone marrow was diluted 2-fold with α-MEM (ICN, Canada) and 0.2 % methylcellulose (1500 cP, Sigma, USA) and left for 40 min at room temperature. The supernatant was collected and precipitated by centrifugation at 450 g for ten minutes. A total of 3 × 10⁶ cells were seeded into a 25 cm² flask (Corning-Costar, USA) containing 5 mL of complete α-MEM nutrient medium (ICN, Canada) supplemented with 10 % fetal bovine serum (FBS; Hyclone, USA), 2 mM l-glutamine (ICN, Canada), 100 U/mL penicillin (Sintez, Russia), and 5 µg/mL streptomycin (BioPharmGarant, Russia). MSCs were cultured at 37°С and 5 % СО_2_. After reaching confluency, the cells were passaged. During passage, 10^5^ cells were seeded in a 25 cm² flask in 5 mL of the medium. Cultures were maintained for four passages.

The MSC population was characterized by flow cytometry. The mean fluorescence intensity (MFI) of fluorescently-labeled antibodies bound to the CD105, CD90, CD73, CD146, CD54 antigens was estimated. All studied MSCs matched the criteria of the International Society of Cellular Therapy.^24^

The time to reach the initial confluence (P0) was defined as the number of days from seeding the bone marrow to reaching confluence for the first time.

Cumulative cell production for three passages was calculated using the following formula:

$$\text{Nsum}=N0+N1*\left(N0+N2\right)/2\;x\;10^{5}+N2*\left(N1+N3\right)/2\;x\;10^{5}$$

where N0, N1, N2, and N3 are the number of cells obtained from 2 culture flasks at passages 0, 1, 2, and 3, respectively.

### Mitochondrial membrane potential and mitochondrial mass assessment

In order to assess mitochondrial membrane potential, the MSCs were stained with tetramethylrhodamine methyl ester perchlorate (TMRM) (Abcam, UK), a potential-based dye that is sequestered by mitochondria and reflects their membrane potential. MitoView Green (Biotium, USA) staining was used to determine relative mitochondrial mass per cell.

The analysis was performed according to the manufacturers’ recommendations using a CytoFLEX flow cytometer (Beckman Coulter, USA). Data were analyzed using the Kaluza Analysis 2.1 program (Beckman Coulter, USA).

In order to characterize mitochondrial activity, the ratio of MFI(TMRM)/MFI(MitoView Green) was calculated.

### RNA and DNA isolation and real-time polymerase chain reaction

The relative gene expression levels (RELs) of MSCs were determined by reverse transcription followed by real-time polymerase chain reaction (PCR) (Taq-Man modification) using the CFX96 Touch Real-Time PCR Detection System (Bio-Rad, USA). RNA was isolated using the Trizol reagent (Life Technologies, USA) according to the standard protocol with minor modifications for the MSCs after the first passage. M-MLV reverse transcriptase (Promega, USA) was used to build the first cDNA strands after RNA hybridization with a mixture of poly-T primers and random hexamers. The housekeeping genes *GAPDH* and *ACTB* were used for normalization. RELs were calculated using the ΔΔCt method.

To determine the ratio of mtDNA and nuclear DNA (nDNA), total DNA was isolated from all MSC samples. Cells were placed in 200 µL of lysing buffer (10 mM TrisHCl pH 7.5, 10 mM EDTA, 10 mM NaCl, 0.5 % w/v sodium lauryl sarcosine, proteinase K 1 mg/mL) for 12 h at 60 °C, DNA was precipitated in the presence of NaCl, ethanol and glycogen.

The NADH dehydrogenase-1 gene (*MT-ND1*) was used as an mtDNA marker, and a non-transcribed region of VISTA hs71 enhancer (LOC110120583) on chromosome 16 was used as a nDNA marker. ΔCt of these genes was obtained by multiplex PCR. The mtDNA/nDNA ratio was calculated using the formula 1.5^ΔCt^, where 1.5 is the primer efficiency in multiplex PCR.^25^

The sequences of primers and probes are presented in Supplementary Table 2. Samples were stored at −20 °C.

### Determination of mtDNA control region sequence

Total DNA was extracted from patient MSCs before and after allo-HSCT and donor leukocytes as described above. The full-length control region (CR), including the most variable region of mtDNA 15967–605 (1208 base pairs), was amplified (Forward-1 primer: CCA TTA GCA CCC AAA GCT, reverse primer: GAT GTG AGC CCG TCT AAA *CA*). After purification by electrophoresis in agarose gel and the Cleanup S-Cap kit (Evrogen, Russia), the primary structure of the amplified fragment was determined using Sanger sequencing (NANOFOR 05, Syntol, Russia) with the forward-1 primer. In case the information obtained was insufficient to distinguish donor and patient mtDNA a forward-2 primer (GCC TAA ATA GCC CAC ACG TT) was used for sequencing. To determine polymorphisms, obtained primary structures were compared to the Cambridge sequence (NC_012920.1), as described by Murakami et al.^26^ The mtDNA sequences of each patient after allo-HSCT were then compared with those of the same patient prior to allo-HSCT and to the respective donor.

## Results

Sequence analysis of mtDNA from MSCs after allo-HSCT did not identify the presence of donor mtDNA in any of the cases even as trace amounts (Table 1 and Supplementary Figure 1). In the work by Golan et al.,^21^ the horizontal mitochondrial transfer occurred both *in vivo* and *in vitro*, although the efficiency was much lower *in vitro*. Moreover, the authors detected horizontal mitochondrial transfer from human CD34^+^ cells to murine MSCs *in vitro*. We attempted to replicate these *in vitro* co-culture experiments. After co-culture of MSCs with healthy donor leukocytes for four days, no donor mtDNA was detected in any of the MSC cultures studied.

**Table 1:** Comparison of the mtDNA sequences from donors and patients before and after allogeneic hematopoietic stem cells transplantation.

Patient-donor group	Cambridge sequence position number	Patient mtDNA before allo-HSCT (MSCs)	Donor mtDNA (leukocytes)	Patient mtDNA after allo-HSCT (MSCs)
1.	16,069 16,126 73 146	C T A T	T C G C	C T A T
2.	16,126 16,153 93	C G A	T A G	C G A
3.	16,093 16,356 16,519 73	C T T A	T C C G	C T T A
4.	16,069 16,163 16,186 16,189 16,294 16,519	C G T C T T	T A C T C C	C G T C T T
5.	16,126 16,163 16,186 16,189 16,294 16,355 16,356 16,519	C G T C T T C C	T A C T C C T T	C G T C T T C C
6.	16,126 73 185 188 228 295 303 462 477 489	T A G A G C C_7_ C C T	C G A G A T C_9_ T T C	T A G A G C C_7_ C C T
7.	16,294 16,296 16,342 16,519	T T T C	C C C T	T T T C
8.	16,298 72 73 152 185 228	C C A T G G	T T G C A A	C C A T G G
9.	16,126 16,129 16,294 16,296 16,304 16,316	C G T T C A	T A C C T G	C G T T C A

In the work by Golan et al.,^21^ mice were lethally irradiated as a pre-transplantation conditioning. Radiation is known to gravely damage stromal cells. Since the patients studied in this work received a reduced intensity conditioning regimen instead, we set out to test whether the difference in conditioning method accounts for the lack of transfer. In order to do that, we co-cultured healthy donor CD45^+^ cells with irradiated MSCs from a third-party healthy donor. However, no horizontal mitochondrial transfer from the leukocytes to the MSCs was detected. Thus, we concluded that the irradiation did not trigger mitochondrial exchange *in vitro*. It appears that human MSCs, despite being impaired by malignant cells, chemotherapy, and pre-transplantation conditioning, do not receive mitochondria from healthy donor HSPCs after allo-HSCT.

Mitochondrial activity affects MSC differentiation and even immunoregulatory properties. When comparing the growth characteristics of MSCs before and after allo-HSCT, no significant differences were found in the cumulative cellular production over three passages nor the proliferation index (Table 2). On the other hand, the time to P0 was significantly longer for MSCs after allo-HSCT.

**Table 2:** Characteristics of mesenchymal stromal cells before and after allogeneic hematopoietic stem cell transplantation.

**MSC characteristic**	**Patient number**	**1**	**2**	**3**	**4**	**5**	**6**	**7**	**8**	**Mean ± SE**	***p*-value**
MSCs for 3 passages	Before allo-HSCT	8.87	17.10	7.98	4.20	12.40	9.76	5.48	7.04	9.10 ± 1.45	**0.81**
	After allo-HSCT	19.20	8.80	6.70	5.95	1.36		4.70	22.80	9.93 ± 2.81	
Time to P0	Before allo-HSCT	10.00	12.00	13.00	12.00	18.00	12.00	10.00	11.00	12.25 ± 0.90	**0.007**
	After allo-HSCT	18.00	14.00	14.00	21.00	20.00	14.00	15.00	14.00	16.25 ± 1.05	
Cell proliferation rate	Before allo-HSCT	7.00	8.60	6.87	7.88	7.79	7.55	5.19	5.70	7.07 ± 0.41	**0.56**
	After allo-HSCT	9.30	7.10	6.80	4.58	1.24		4.93	9.43	6.20 ± 1.02	
REL of *PGC1A*	Before allo-HSCT	0.01	0.00	0.03	0.02	0.01	0.01	0.00	0.00	0.01 ± 0.00	**0.34**
	After allo-HSCT	0.28	0.04	0.01	0.00	0.02		0.02	0.01	0.05 ± 0.04	
REL of *NFE2L2*	Before allo-HSCT	1.59	1.37	2.02	3.21	2.80	0.93	1.57	2.08	1.94 ± 0.27	**0.37**
	After allo-HSCT	3.70	2.84	2.10	1.99	2.17	0.57	3.02	2.37	2.35 ± 0.33	
REL of *NQO1*	Before allo-HSCT	2.63	2.15	1.13	0.59	1.52	0.70	3.46	3.40	1.95 ± 0.40	**0.72**
	After allo-HSCT	1.08	1.32	1.64	1.75	3.93	0.56	1.86	1.88	1.75 ± 0.35	
REL of *HO1*	Before allo-HSCT	4.70	3.56	3.99	3.45	4.18	4.47	4.30	10.95	4.95 ± 0.87	**0.78**
	After allo-HSCT	9.09	6.61	4.59	3.64	10.13	0.61	3.38	4.88	5.37 ± 1.11	
REL of *GCLC*	Before allo-HSCT	1.87	1.61	4.06	4.23	2.77	1.60	2.94	2.77	2.73 ± 0.36	**0.59**
	After allo-HSCT	5.09	2.09	3.08	2.74	2.08		4.72	2.95	3.25 ± 0.42	
REL of *HIF1A*	Before allo-HSCT	1.16	0.82	0.27	1.78	0.73	0.62	0.70	1.51	0.95 ± 0.18	**0.21**
	After allo-HSCT	2.10	1.18	0.97	0.96	1.28		1.29	1.43	1.32 ± 0.14	
REL of *LDHA*	Before allo-HSCT	2.74	2.58	0.38	1.41	0.84	2.58	3.00	2.27	1.97 ± 0.34	**0.56**
	After allo-HSCT	1.24	1.27	1.49	2.94	2.28	0.07	1.85	1.98	1.64 ± 0.30	
mtDNA/nDNA	Before allo-HSCT	21.92	56.26	67.04	15.98	6.49	2.76	20.98	6.08	24.69 ± 8.50	**0.082**
	After allo-HSCT	6.97	6.26	15.20	13.84	3.20	3.78	6.90	11.79	8.49 ± 1.61	

SE: standard error; REL: relative gene expression level; MSC: mesenchymal stromal cell; allo-HSCT: allogeneic hematopoietic stem cell transplantation; P0: initial confluence.

This study analyzed the RELs of genes regulating mitochondrial biogenesis and metabolic activity and the ratio of mtDNA/nDNA (Table 2).

## Discussion

**Figure 2:**
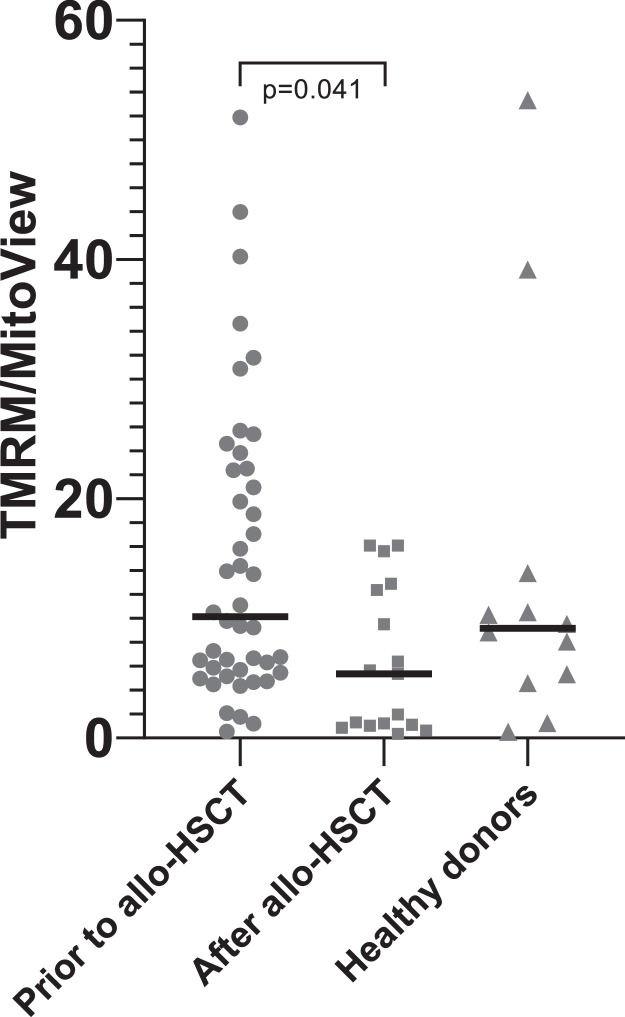
The mean fluorescence intensity (MFI) tetramethylrhodamine methyl ester perchlorate (TMRM) to MFI MitoView green ratio. To assess mitochondrial membrane potential, MSCs were stained with TMRM. MitoView green staining was used to determine relative mitochondrial mass per cell. Mitochondrial activity, characterized by the MFI(TMRM)/MFI(MitoView Green) ratio, was found to be significantly decreased after allo-HSCT.

Treatment of patients with acute leukemia is associated with intensive chemotherapy courses that not only kill tumor and hematopoietic cells, but also damage the stromal microenvironment of the bone marrow.^27^ After allo-HSCT, which involves conditioning the recipient, all recipient hematopoietic cells are eliminated, and stromal progenitor cells are damaged even more. Thus, the quality of hematopoiesis worsens. The murine model data that transplantation of healthy HSPCs can lead to the transfer of mitochondria from healthy cells to damaged MSCs were very attractive. This study attempted to determine whether such a transfer occurs after the restoration of hematopoiesis in patients. Contrary to data from murine models,^23^^,^^21^ mitochondrial transfer was not detected from transplanted HSPCs to recipient MSCs in patients after allo-HSCT. In addition, the main properties of the patient MSCs both before and after allo-HSCT were studied. The only statistically significant difference was an increase in the time to P0 after allo-HSCT, indicating a decrease in the concentration of MSCs in the bone marrow. The mtDNA/nDNA ratio in MSCs of six out of eight patients studied decreased after allo-HSCT (Table 2), which confirms the data of other authors on mitochondrial damage after radiation and chemotherapy.^21^^,^^28^ Additionally, in a larger cohort of patients, mitochondrial activity decreased after allo-HSCT, as evidenced by a decrease in the TMRM/MitoView ratio (Figure 2).

In their quiescent state, MSCs seem to favor glycolysis, whereas during proliferation their metabolism shifts to rely more on mitochondrial activity.^29^ RELs of genes regulating mitochondrial biogenesis (*PGC1A, NFE2L2* and *HIF1A*),^30^ associated with oxidative stress (*NQO1, HO1, GCLC*),^31^ and regulating anaerobic glycolysis (*LDHA*) did not differ significantly in MSCs before and after allo-HSCT (Table 2). Thus, the data of this study demonstrate that HSPC transplantation from a healthy donor does not promote MSC metabolism.

The absence of donor mitochondrial markers in recipient MSCs after allo-HSCT and changes in the expression of genes responsible for mitochondrial metabolism indicate the absence of mitochondrial transfer from transplanted healthy HSPCs to recipient mesenchymal cells. In this work, no methods were used to label potentially transferred Mt, except the natural mitochondrial DNA markers. As has been described, dye markers may distort the results.^14^ In studies on mice, sorted cells were used and mitochondrial markers were examined in the total bone marrow cell population.^23^ In this study, recipient MSCs were studied after allo-HSCT without any additional processing. It has been shown previously that the proteome of MSCs from patients at the onset and remission of acute leukemia lacks many proteins associated with mitochondrial biogenesis, which are present in MSCs from donors.^32^ One could expect an improvement in the metabolic status of MSC mitochondria after allo-HSCT, but this did not happen.

## Conclusion

Based on the data obtained, one cannot count on an improvement in the stromal microenvironment due to horizontal mitochondrial transfer from healthy donor HSPCs to patient MSCs after transplantation. It is obvious that the stromal microenvironment after allo-HSCT is severely damaged and the development of new therapies is required to improve its condition.

## Conflicts of interest

The authors declare no conflicts of interest.

## Appendix Supplementary materials

Image, application 1
