# Supplementary material for: There is no transfer of mitochondria from donor hematopoietic cells to recipient mesenchymal stromal cells after allogeneic hematopoietic stem cells transplantation in humans
Source: Hematol Transfus Cell Ther. 2025 Jun 18;47(3):103859. doi: 10.1016/j.htct.2025.103859 (PMC12209926; doi:10.1016/j.htct.2025.103859)
Supplement: Supplementary file 1 [file mmc1.docx]

Supplementary data

**Supplementary Table 1:** Patient characteristics

| Patient number | Leukemia variant | Age, years | Gender | Donor |
| --- | --- | --- | --- | --- |
| 1 | ALL | 27 | Male | unrelated compatible |
| 2 | ALL | 29 | Male | haploidentical father |
| 3 | ALL | 20 | Female | unrelated compatible |
| 4 | AML | 47 | Female | unrelated compatible |
| 5 | AML | 29 | Male | unrelated compatible |
| 6 | ALL | 24 | Male | unrelated partially compatible |
| 7 | AML | 28 | Female | unrelated compatible |
| 8 | AML | 21 | Female | unrelated compatible |
| 9 | ALL | 48 | Male | unrelated compatible |

ALL: Acute lymphoblastic leukemia; AML: Acute myeloid leukemia

**Supplementary Table 2:** Nucleotide sequences of real-time polymerase chain reaction primers and probes

| **Gene** | **Purpose** | **Sequence - 5’ to 3’** |
| --- | --- | --- |
| *ACTB* | Forward primer | CAACCGCGAGAAGATGACC |
| *ACTB* | Reverse primer | CAGAGGCGTACAGGGATAGC |
| *ACTB* | Probe | ROX-AGACCTTCAACACCCCAGCCATGTACG-BHQ2 |
| *GAPDH* | Forward primer | GGTGAAGGTCGGAGTCAACG |
| *GAPDH* | Reverse primer | TGGGTGGAATCATATTGGAACA |
| *GAPDH* | Probe | ROX-CTCTGGTAAAGTGGATATTGTTGCCATCA-BHQ2 |
| *PGC1A* | Forward primer | ATTTGAGAACAAGACTATTGAAC |
| *PGC1A* | Reverse primer | AGGGTTATCTTGGTTGGCTTT |
| *PGC1A* | Probe | FAM-CTTAAGTGTGGAACTCTCTGGAACT-RTQ1 |
| *NFE2L2* | Forward primer | CAGTTACAACTAGATGAAGAGA |
| *NFE2L2* | Reverse primer | CGGGAATATCAGGAACAAGT |
| *NFE2L2* | Probe | ROX-CAAAAGCTGCATGCAGTCATCAAAG-BHQ2 |
| *PGC1A* | Forward primer | ATTTGAGAACAAGACTATTGAAC |
| *PGC1A* | Reverse primer | AGGGTTATCTTGGTTGGCTTT |
| *PGC1A* | Probe | FAM-CTTAAGTGTGGAACTCTCTGGAACT-RTQ1 |
| *HIF1A* | Forward primer | AATTTTGATCCCCTTTCTACTTAA |
| *HIF1A* | Reverse primer | CTACTGCAATGCAATGGTTTAA |
| *HIF1A* | Probe | FAM-TTTTAGTATGTTCTTTAATGCTGGATCA-RTQ1 |
| *NQO1* | Forward primer | ACCTCTATGCCATGAACTTCA |
| *NQO1* | Reverse primer | CCTTCTTTATAAGCCAGAACA |
| *NQO1* | Probe | FAM-CCAGAAAGGACATCACAGGTAAACT-RTQ1 |
| *HO1* | Forward primer | AGCAACAAAGTGCAAGATTCT |
| *HO1* | Reverse primer | CATAAAGCCCTACAGCAACT |
| *HO1* | Probe | FAM-ACTCAACACCCGCTCCCAGGCT-RTQ1 |
| *GCLC* | Forward primer | CTACTATCTGTCCAATTGTTATG |
| *GCLC* | Reverse primer | CCGAGTTCTATCATCTACAGA |
| *GCLC* | Probe | FAM-CTTTTACCGAGGCTATGTGTCAGAC-RTQ1 |
| *LDHA* | Forward primer | GAGATTCCAGTGTGCCTGTAT |
| *LDHA* | Reverse primer | TTGTGAACCTCTTTCCACTGT |
| *LDHA* | Probe | FAM-TCTTCAGAGAGACACCAGCAACATT-RTQ1 |
| *MT-ND1* | Forward primer | TAGCCTAGCCGTTTACTCAAT |
| *MT-ND1* | Reverse primer | TTAGTAATGTTGATAGTAGAATG |
| *MT-ND1* | Probe | ROX-CATATGAGATTGTTTGGGCTACTGC-BHQ2 |
| *GENE CONT* | Forward primer | TCCACGCGCCTCAACCTA |
| *GENE CONT* | Reverse primer | GATAATTGAGCCAGCCTCTG |
| *GENE CONT* | Probe | FAM-CCTGAAGGATAAGATACTCGAAATGCCAC-RTQ1 |


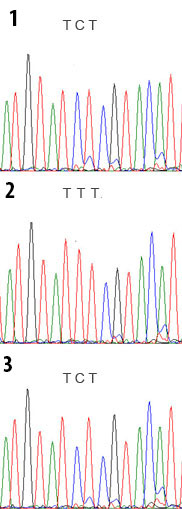


**Supplementary Figure 1:** 1) An example of mitochondrial DNA sequences obtained from mesenchymal stromal cells (MSCs) prior to allogeneic hematopoietic stem cell transplantation (allo-HSCT): 2) Respective donor lymphocytes: 3) MSCs from the same patient after allo-HSCT
